# Concurrent Bartholin gland and perianal abscesses in a woman: a case report of a diagnostic challenge

**DOI:** 10.3389/fmed.2026.1982913

**Published:** 2026-10-09

**Authors:** Jiansheng Hu, Liyun Dong, Linmei Sun, Hongbo Su

**Affiliations:** 1 Department of Anorectal Surgery, Xi'an Hospital of Traditional Chinese Medicine, Xi'an, Shaanxi, China; 2 Department of Gynecology, Xi'an Hospital of Traditional Chinese Medicine, Xi'an, China

**Keywords:** Bartholin gland abscess, case report, concomitant infection, perianal abscess, perineal infection

## Abstract

**Background:**

Bartholin gland abscess and perianal abscess are common suppurative infections involving anatomically adjacent regions of the female perineum. Because both conditions may present with localized pain and swelling, distinguishing their respective origins and determining the extent of infection can be challenging. The concomitant occurrence of these two abscesses is uncommon, and its diagnostic and therapeutic considerations remain poorly characterized. We report a rare case of concomitant Bartholin gland and perianal abscesses and describe the diagnostic process and multidisciplinary surgical management.

**Case presentation:**

An 18-year-old woman presented with progressive perineal and perianal pain and swelling. Pelvic computed tomography demonstrated abscess formation involving the perianal and left perineal soft tissues, and a possible communication between the two lesions was initially suspected. Emergency surgery was performed. The perianal abscess was incised and drained, and an infected anal gland was identified and excised, with bridge drainage established between the drainage incisions. Intraoperative exploration revealed no communication between the perianal abscess and the adjacent perineal lesion. Following gynecological consultation, the separate left vulvar abscess was drained and treated with Jacobi ring fistulization. Culture of the perineal abscess grew Escherichia coli, whereas the perianal abscess culture showed no bacterial growth. Follow-up magnetic resonance imaging 1 month after surgery demonstrated a cystic lesion in the anatomical region of the left Bartholin gland, supporting the Bartholin gland origin of the previously drained perineal abscess. The patient showed satisfactory clinical recovery, with complete healing of the perianal and perineal wounds at 4 months and no evidence of recurrent abscess during the available follow-up.

**Conclusion:**

Concomitant Bartholin gland and perianal abscesses may be difficult to distinguish because of their overlapping clinical manifestations and anatomical proximity. Clinicians should consider the possibility of multiple independent or potentially related infectious foci and systematically evaluate adjacent anatomical regions. Careful physical examination, appropriate imaging, intraoperative assessment, and timely multidisciplinary collaboration can facilitate accurate diagnosis and individualized surgical management.

## 1 Introduction

The Bartholin glands, also known as the greater vestibular glands, are located bilaterally at the posterolateral aspect of the vaginal introitus and primarily secrete mucus that contributes to vulvovaginal lubrication (1). Obstruction of the Bartholin gland duct can lead to secondary infection and abscess formation (2). Bartholin gland abscess is a relatively common condition among women of reproductive age, with an estimated annual incidence of approximately 0.95 ± 0.06 per 1,000 women (3). The infection is often polymicrobial and is predominantly caused by opportunistic bacteria, although sexually transmitted pathogens may be identified in a minority of cases (3). Recurrence is clinically relevant, occurring in approximately one-third of patients after treatment (4). Typical manifestations include localized vulvar swelling and pain, which can be severe enough to interfere with daily activities; spontaneous rupture may occur in some cases as the inflammatory process progresses (5). Once an abscess has developed, adequate drainage and maintenance of a drainage tract are the key principles of treatment. Various approaches have been described, including incision and drainage, marsupialization, Word catheter placement, Jacobi ring fistulization, carbon dioxide laser treatment, and silver nitrate cauterization. Compared with simple incision and drainage, procedures that maintain continuous drainage, such as catheter placement or fistulization, may reduce the risk of recurrence (6).

**Table 1:** Clinical timeline of the patient's presentation, diagnostic evaluation, surgical management, and follow-up.

Time	Clinical events
15 days before admission	Perineal pain and swelling developed
1 day before admission	Perineal symptoms worsened, with acute severe perianal pain and swelling.
Apr 20, 2026	Admission; CT revealed perianal and perineal abscesses. Emergency surgery was performed, including perianal abscess drainage, removal of the infected anal gland tissue as the presumed source of infection, and Jacobi ring fistulization of the adjacent vulvar abscess. No communication between the two abscesses was identified intraoperatively.
Apr 27, 2026	One-week postoperative MRI showed postoperative inflammatory changes without a definite residual abscess.
Apr 30, 2026	The patient was discharged on postoperative day 10.
May 27, 2026	One-month MRI identified a cystic lesion in the left Bartholin gland region, supporting the diagnosis of Bartholin gland abscess.
July 19 to August 27, 2026	At 4-month follow-up, the perianal and perineal wounds had completely healed, with no evidence of recurrent abscess; occasional vaginal discharge persisted.

Perianal abscess is another common suppurative infection of the anorectal region, most often resulting from infection of the anal glands. Patients typically present with perianal pain and swelling, with fever or systemic symptoms occurring in some cases. Although superficial abscesses may be readily identified by physical examination, deep abscesses involving the ischiorectal fossa or pelvic spaces can present with nonspecific perineal, pelvic, or lumbosacral pain. Imaging modalities, including computed tomography, magnetic resonance imaging, and endorectal ultrasonography, may therefore be required to delineate the location and extent of infection and its relationship to adjacent structures (7, 8). Once diagnosed, prompt surgical drainage is generally required to achieve adequate source control and prevent progression to adjacent tissues or systemic infection.

Although Bartholin gland abscess and perianal abscess are both common suppurative conditions, their concomitant occurrence is uncommon and has rarely been reported. Because these infections arise in anatomically adjacent regions and may share similar clinical manifestations, including perineal pain and swelling, distinguishing their respective origins and determining the full extent of infection can be challenging. In particular, a Bartholin gland abscess may be mistaken for an adjacent perianal abscess or fistulous disease, potentially resulting in incomplete drainage or delayed treatment. Careful examination of both the vulvar and perianal regions, supplemented by appropriate imaging when necessary, is therefore important in patients with overlapping perineal infections. In complex cases, collaboration between gynecologists and colorectal surgeons may facilitate accurate diagnosis and coordinated surgical management.

Here, we report a rare case of concomitant Bartholin gland and perianal abscesses in a female patient. The perianal abscess was managed by incision and drainage with intraoperative treatment of the identified internal opening, whereas the Bartholin gland abscess was treated with Jacobi ring fistulization. This case highlights the diagnostic challenges posed by anatomically overlapping perineal infections and the potential value of multidisciplinary assessment and individualized surgical management.

## 2 Case presentation

### 2.1 Patient information and initial presentation

An 18-year-old woman (body mass index, 19.1 kg/m^2^) was admitted with a 15-day history of perineal pain and swelling and acute worsening of perianal pain and swelling during the preceding day. She was a student, unmarried, and nulligravid. She had no known history of diabetes mellitus, immunodeficiency, autoimmune disease, or other relevant chronic conditions.

Approximately 15 days before admission, she developed mild, nonspecific pain and swelling in the perineal region without an identifiable precipitating factor. She did not seek medical attention or receive treatment at that time. One day before admission, the perineal symptoms progressively worsened, and she developed severe perianal pain and swelling. She initially presented to a local hospital, where intravenous antimicrobial therapy was administered; however, her symptoms did not improve. At approximately 1:00 a.m., she was urgently transferred to Xi'an Hospital of Traditional Chinese Medicine and admitted through the emergency department, accompanied by her family.

On admission, she appeared uncomfortable and was in moderate distress because of severe perianal pain. Her body temperature was 37.2 °C, pulse rate was 133 beats/min, respiratory rate was 18 breaths/min, and blood pressure was 97/63 mmHg. She reported difficulty with defecation but no urinary symptoms.

Physical examination revealed marked swelling of the perineal and perianal regions, with the vulvar and perianal lesions located in close anatomical proximity. A swollen area measuring approximately 5 × 3 cm was present in the lower portion of the left vulva. The overlying skin was intact, with no evidence of spontaneous rupture. A small amount of purulent discharge was observed at the vaginal introitus. Additionally, a tender mass measuring approximately 5 × 4 cm was palpable in the left anterior perianal region, extending from the 12 to 3 o’clock positions in the lithotomy position. The lesion corresponded to an abscess involving the left ischiorectal fossa and intersphincteric space, as confirmed by imaging and intraoperative findings. The overlying skin showed marked erythema, increased local temperature, and pronounced tenderness, without visible rupture. Digital rectal examination revealed elevation of the anal canal mucosa extending from approximately the 12 to 5 o'clock positions. No blood was observed on the examining glove. A detailed timeline of the patient's presentation, diagnostic evaluation, surgical management, and follow-up is provided in Table 1.

### 2.2 Diagnostic assessment

Initial laboratory investigations revealed a white blood cell count of 13.8 × 10⁹/L, with neutrophils accounting for 80% of leukocytes. The C-reactive protein level was elevated at 68.3 mg/L, whereas the procalcitonin level was 0.06 ng/mL.

Pelvic computed tomography (CT) without contrast enhancement demonstrated marked thickening and swelling of the perianal and left perineal soft tissues. A localized low-density fluid collection was present within the affected soft tissues, accompanied by surrounding fat stranding and blurred fat planes, suggesting localized suppurative infection with abscess formation (Figure 1). Given the severity of the infection and the need for urgent surgical intervention, contrast-enhanced CT and Magnetic Resonance Imaging (MRI) were not performed to avoid delaying definitive surgical management. Based on the clinical findings and CT appearance, a perianal abscess with an adjacent vulvar/perineal abscess was initially suspected. Because the two lesions were anatomically adjacent, a possible infectious connection between them was considered before surgery.

**Figure 1:**
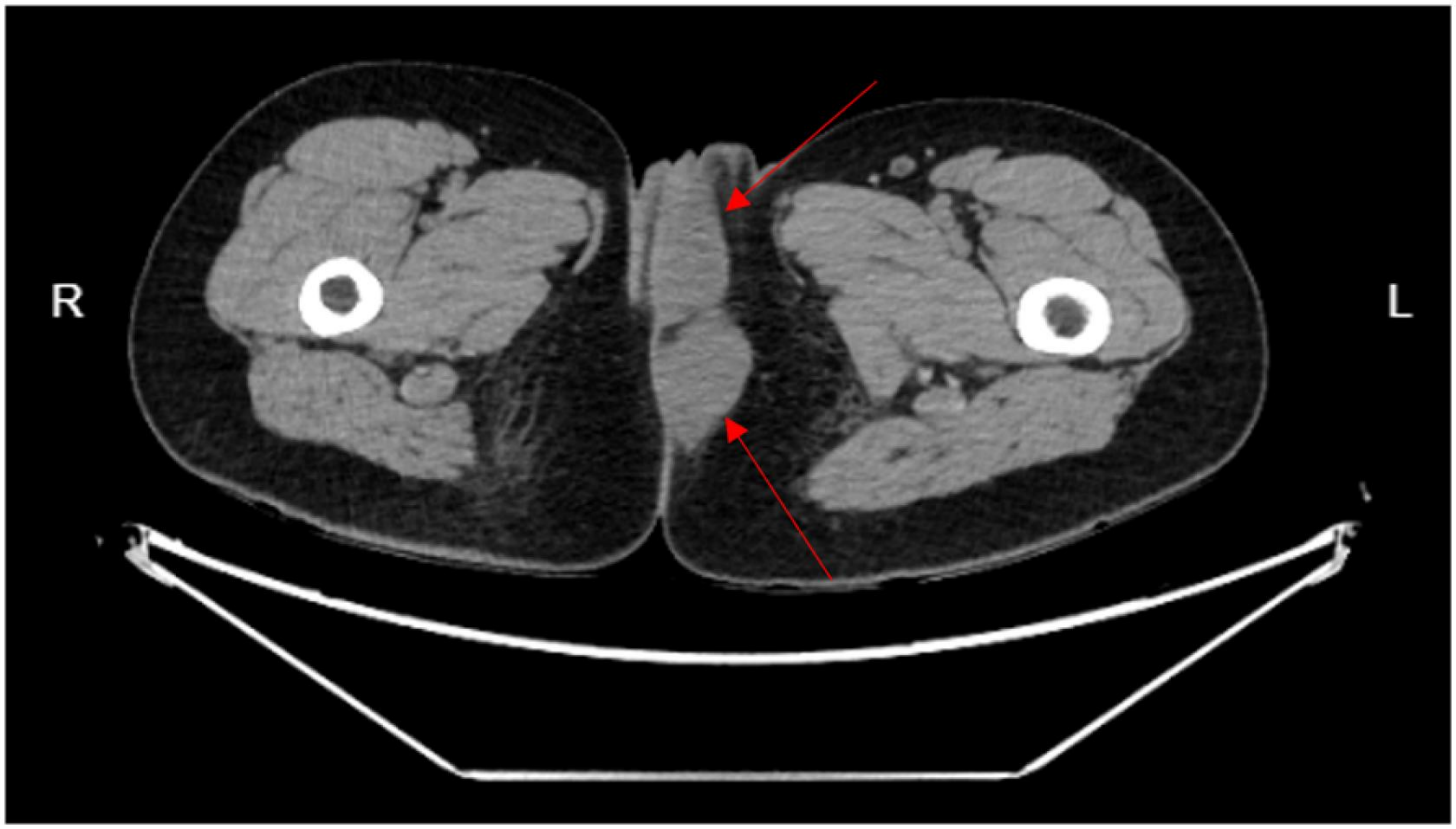
Axial CT image showing diffuse soft-tissue swelling and inflammatory changes involving the left perianal and vulvar regions.

Given the severity of the perianal symptoms and the imaging evidence of abscess formation, urgent surgical drainage was considered necessary. The definitive relationship between the perianal and perineal lesions was assessed intraoperatively.

### 2.3 Therapeutic intervention and intraoperative findings

The patient was admitted at 00:55 on April 20, 2026. After completion of the necessary laboratory and imaging evaluations, emergency surgical drainage was performed at 02:55 under spinal anesthesia.

The patient was placed in the lithotomy position. After routine disinfection and sterile draping, the anal canal was disinfected three times with povidone-iodine.

A radial incision was first made at the 1 o'clock position, approximately 4 cm from the anal verge. The skin and subcutaneous tissues were incised, and blunt dissection with curved forceps was used to enter the abscess cavity. Approximately 10 mL of blood-tinged purulent material was drained, and a specimen was collected for bacterial culture. Digital exploration demonstrated that the abscess cavity extended from the 1 o'clock position toward the 6 o'clock position, involving the intersphincteric and external sphincter regions and extending into the right ischiorectal fossa.

A second radial incision was therefore made at the 6 o’clock position, approximately 3 cm from the anal verge. The abscess cavity was opened, and the cavity wall was gently debrided with gauze. Exploration through the 6 o’clock incision demonstrated extension of the cavity along the intersphincteric plane. Following the direction of the involved tissue, dissection was continued toward the anal crypt, where the infected anal gland tissue at the 6 o’clock position was identified as the presumed source of infection and excised. The tissues on both sides of the involved gland were ligated. A loose rubber-band drain was placed between the 1 and 6 o’clock incisions to establish bridge drainage and maintain continuous drainage of the abscess cavity.

The perianal abscess cavity was then explored toward the perineal region through the 1 o'clock incision. No communication between the perianal abscess cavity and the adjacent perineal lesion was identified. Because a separate and persistent perineal lesion remained, an intraoperative gynecological consultation was requested.

The gynecologist performed a systematic examination of the vulvar and perineal regions. No communication between the perianal and perineal surgical sites was identified. During the examination, a small amount of thin purulent discharge was intermittently observed emerging from the vaginal introitus. A specimen of the discharge was collected for bacterial culture and testing for Mycoplasma, Chlamydia, and Neisseria gonorrhoeae.

After repeat disinfection of the perineal region, a well-defined, fluctuant mass measuring approximately 5.0 × 3.0 × 2.0 cm was palpated in the middle and lower portion of the left labium majus. The overlying skin was erythematous and warm, and there was no spontaneous rupture. The labium minus was everted, and a 0.5-cm incision was made at the most dependent and fluctuant portion of the lesion at the skin–mucosal junction. A large amount of gray-yellow purulent material was immediately drained. Under the guidance of a hemostatic forceps, a second small incision was created at the opposite end of the abscess cavity. A rubber strip was passed through the entire cavity and its two ends were tied together to form a closed ring, thereby establishing a Jacobi ring fistulization tract. The cavity was repeatedly irrigated with normal saline, and no active bleeding was observed.

After adequate hemostasis and drainage had been confirmed, petrolatum gauze was placed within the wound cavity, followed by a pressure dressing. The patient was transferred to the ward in stable condition.

### 2.4 Microbiological and pathological findings

Purulent specimens were collected separately from the perianal abscess and the perineal abscess during surgery. Bacterial culture of the perianal abscess specimen showed no bacterial growth. In contrast, culture of the purulent material obtained from the perineal abscess yielded *Escherichia coli* (+++, moderate growth). Antimicrobial susceptibility testing showed susceptibility to multiple tested antimicrobial agents, with resistance or intermediate susceptibility to several other agents.

Samples obtained from the perineal abscess were additionally submitted for testing for Mycoplasma, Chlamydia, and Neisseria gonorrhoeae. However, the amount of purulent material obtained intraoperatively was insufficient for valid testing, and no definitive results were available.

Histopathological examination of tissue obtained from the perianal lesion demonstrated extensive inflammatory cell infiltration, predominantly composed of neutrophils, together with tissue necrosis and purulent exudate formation. Granulation tissue proliferation, capillary dilation, and fibrous tissue formation were observed in the surrounding tissue. These findings were consistent with acute suppurative inflammation (Figure 2).

**Figure 2:**
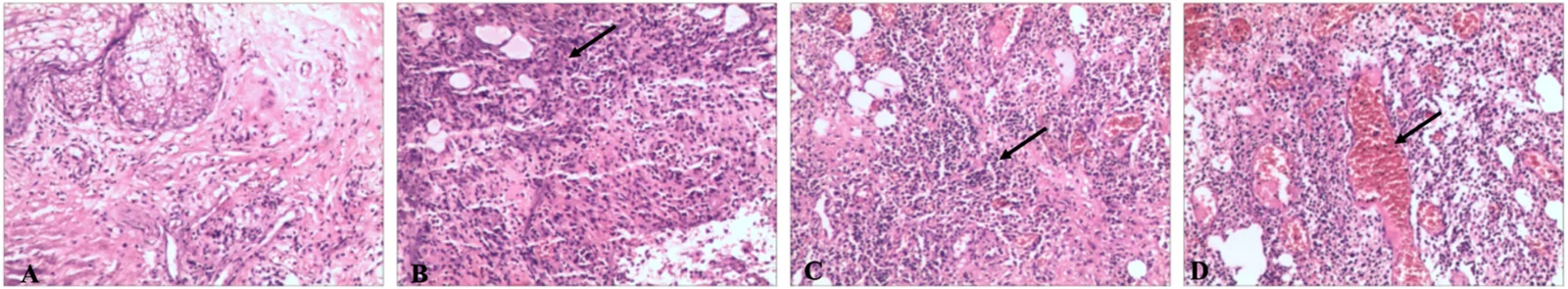
Pathological findings of necrotic tissue from perianal abscess. **(A)** Histopathological examination showing inflammatory changes in the affected tissue. **(B)** Prominent inflammatory cell infiltration within the soft tissue (arrow). **(C)** Dense inflammatory cell infiltration associated with marked tissue inflammation (arrow).**(D)** Focal tissue necrosis surrounded by inflammatory cell infiltration, with the arrow indicating the necrotic area.

The patient had received a short course of intravenous antimicrobial therapy at the referring hospital before transfer because of the acute worsening of her symptoms. After admission and surgical drainage, intravenous fosfomycin sodium was continued for a total of 5 days as perioperative antimicrobial therapy. Five days after treatment, her complete blood count had returned to within the normal range, and her local symptoms and inflammatory manifestations had substantially improved. No further systemic signs of infection were observed.

### 2.5 Early postoperative course and follow-up

One week after surgery, both the perianal and perineal wounds were healing appropriately. The rubber-band drain remained in place and functioned effectively. However, a relatively large amount of thin discharge continued to emerge intermittently from the vaginal introitus.

Perianal MRI performed on April 27, 2026, demonstrated marked edema of the perianal and perineal soft tissues, with patchy and linear high-signal intensities on T2-weighted images and inflammatory exudation in the operative region. The local tissue architecture remained distorted, with inflammatory infiltration of the soft tissues surrounding the anal canal and rectum. No definite residual localized abscess cavity was identified. These findings were considered compatible with early postoperative inflammatory changes and tissue repair (Figure 3).

**Figure 3:**
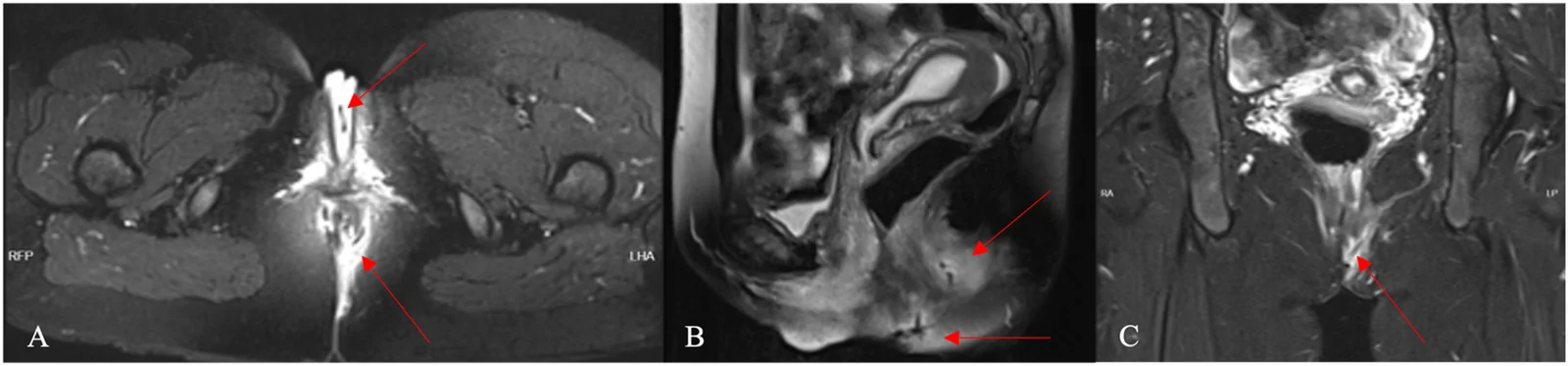
Perianal MRI findings one week after surgery. **(A)** Axial image showing marked postoperative inflammatory changes and soft-tissue edema involving the perineal and labial regions (arrows). **(B)** Sagittal image showing residual inflammatory changes in the perianal soft tissues (arrows). **(C)** Coronal image demonstrating persistent inflammatory edema in the perineal and labial regions (arrow). Although the infection was clinically controlled, the extensive local inflammatory edema limited evaluation of the Bartholin gland region, and the presence or absence of a residual Bartholin cyst could not be reliably determined at this time point.

At 1 month after surgery, the perianal and perineal wounds had healed satisfactorily. The patient reported occasional small amounts of thin discharge from the vaginal introitus after physical activity. Perianal MRI performed on May 27, 2026, demonstrated postoperative changes involving the anal canal and perianal soft tissues, with patchy inflammatory high-signal intensity and partially obscured tissue planes. No definite encapsulated fluid collection or complete continuous fistulous tract was identified. Importantly, a well-circumscribed, round cystic lesion with homogeneous high signal intensity on T2-weighted images was observed in the region of the left Bartholin gland (Figure 4). The lesion was located in the posterolateral aspect of the left labium majus, corresponding anatomically to the Bartholin gland region, without an obvious solid component or surrounding tissue infiltration. These findings supported the anatomical origin of the previously drained perineal abscess from the left Bartholin gland region.

**Figure 4:**
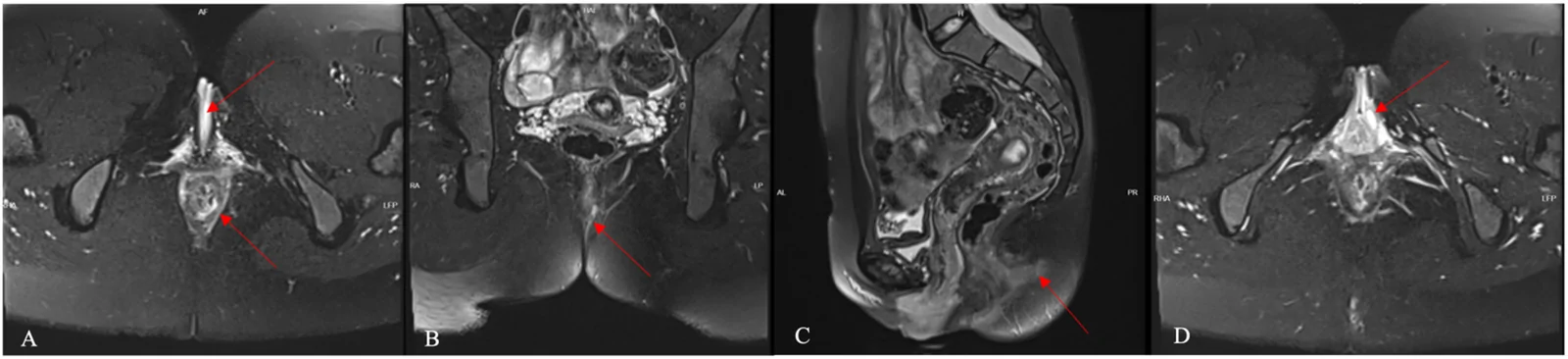
Perianal MRI scan at 1-month post-operation. **(A–C)** Axial, coronal, and sagittal images demonstrate progressive healing of the postoperative wound, with residual inflammatory changes and soft-tissue edema in the perianal and perineal regions (arrows). No definite fistulous tract was identified. **(D)** Axial image demonstrating the Bartholin cyst (arrow).

Vaginal microbiological evaluation performed on July 19, 2026, was negative for bacterial vaginosis, Trichomonas vaginalis, and fungal hyphae. Fungal spores were observed, while vaginal lactobacilli were present only in trace amounts (±). The white blood cell count in the vaginal secretion was >30 cells/high-power field, and the cleanliness grade was IV, indicating marked disruption of the vaginal microbiota and nonspecific vaginal inflammation (Table 2).

**Table 2:** Results of vaginal microbiota analysis.

Test Item	Result	Reference range	Unit
Rapid test for bacterial vaginosis	Negative	Negative	
Clue cells	Negative	Negative	
Trichomonas-like flagellated bacteria	Negative	Negative	
Cocci	Negative	Negative	
*Gardnerella vaginalis*	Negative	Negative	
Trichomonas	Not found	Negative or not found	
Molds	Spores	Negative	
Lactobacillus (vaginal bacilli)	±	++∼++++	
Epithelial cells	5-8	++∼++++	/HP
Leukocytes	>30	None∼+	/HP
Vaginal cleanliness grade	IV	I∼II	Grade

At the latest outpatient follow-up on August 27, 2026, focused physical examination demonstrated satisfactory healing of the perianal and perineal surgical wounds without evidence of recurrence. The patient continued to report intermittent scant vaginal discharge, which did not interfere with her daily activities or studies. Follow-up MRI demonstrated a marked reduction in the size of the Bartholin gland cyst (red arrow) compared with the preoperative findings (Figure 5).

**Figure 5:**
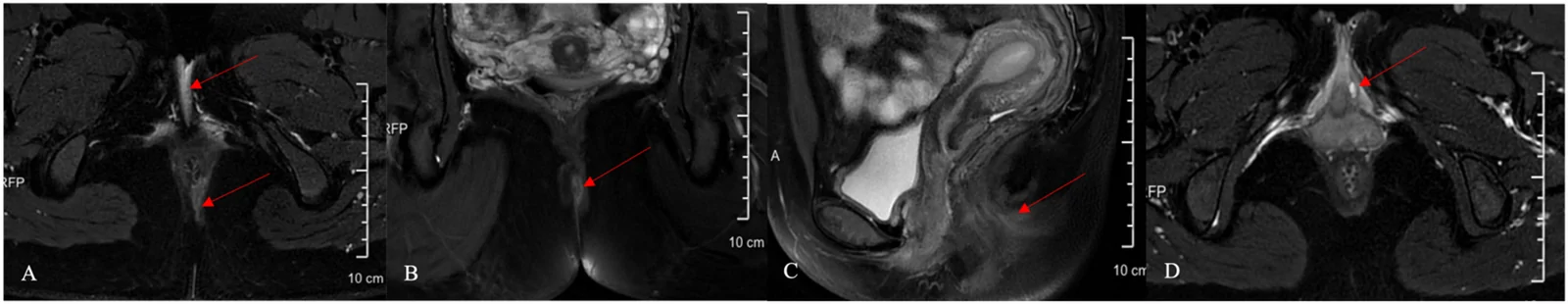
Perianal MRI scan at 4-month post-operation. **(A–C)** Axial, coronal, and sagittal images show postoperative fibrotic scar tissue at the previous surgical site in the perianal and perineal region (arrows), consistent with wound healing. The surrounding inflammatory changes have markedly resolved. **(D)** Axial image shows a residual Bartholin gland cyst (arrow), which has markedly decreased in size compared with the MRI obtained one month after surgery.

Overall, the patient showed satisfactory postoperative recovery. At the 4-month follow-up, the perianal wound had completely healed, although occasional minimal discharge from the perineal area persisted, with no evidence of recurrent abscess formation during the follow-up period.

## 3 Discussion

Bartholin gland abscess is a common vulvar infectious condition among women of reproductive age (9). The Bartholin glands are located posterolaterally to the vaginal vestibule, with ductal openings typically located at the 4- and 8-o'clock positions on either side of the vaginal introitus. Obstruction of the relatively narrow duct may result from local edema, inflammation, or mechanical factors, leading to cyst formation and, subsequently, abscess formation after secondary infection (5, 10, 11). The microbiology of Bartholin gland abscesses is usually polymicrobial or dominated by opportunistic pathogens, with Escherichia coli among the organisms most frequently isolated, whereas sexually transmitted pathogens account for a relatively small proportion of cases (12). Neisseria gonorrhoeae and Chlamydia trachomatis have nevertheless been detected in some Bartholin gland abscesses and cervical specimens (13). Therefore, testing for sexually transmitted infections should be guided by the patient's clinical manifestations, epidemiological risk factors, and sexual history rather than performed solely because a Bartholin gland abscess is present. Recurrence is also clinically relevant and tends to occur on the same side in some patients (12, 14).

Although Bartholin gland abscesses are generally confined to the vulvar region, extension into adjacent tissues or the development of complex fistulous disease has occasionally been reported. Perineal necrotizing fasciitis following drainage of a Bartholin gland abscess and recurrent rectovaginal fistula associated with Bartholin gland inflammation have both been described (15, 16). These reports indicate that severe or complicated infection, although uncommon, may extend beyond the expected anatomical boundaries. In the present case, the simultaneous presence of a left vulvar/perineal abscess and a perianal abscess created an additional diagnostic challenge because the two lesions were anatomically adjacent and produced overlapping symptoms.

Perianal abscesses are most commonly associated with infection of the anal glands and subsequent spread into the surrounding tissue spaces (17). In the present case, preoperative clinical assessment and CT suggested the presence of adjacent perianal and perineal abscesses, and a possible connection between the two lesions was initially considered. However, intraoperative exploration demonstrated that the perianal abscess cavity did not communicate with the adjacent perineal lesion. An infected anal gland was identified at the 6-o'clock position and treated, while the separate left vulvar/perineal abscess was independently drained and managed by Jacobi ring fistulization. These findings did not demonstrate a direct anatomical connection between the two abscesses and therefore did not support direct extension of the perianal abscess into the vulvar/perineal lesion. Subsequent MRI demonstrated a cystic lesion in the anatomical region of the left Bartholin gland, providing further support for a Bartholin gland origin of the previously drained perineal abscess. In patients with recurrent or complex perianal abscesses, Crohn's disease should be considered when clinically indicated. In the present case, there were no clinical or imaging findings suggestive of underlying inflammatory bowel disease.

Reports describing the coexistence of Bartholin gland abscess and perianal abscess are rare. Previous studies have mainly focused on isolated Bartholin gland infections or their local complications. In this case, the two abscesses occurred simultaneously without an identifiable anatomical connection, highlighting the importance of evaluating multiple infectious foci in patients with adjacent perineal abscesses. The relationship between the two infectious foci remains uncertain. The anatomical proximity of the Bartholin gland and perianal region provides a theoretical possibility of spread along contiguous soft-tissue planes. Local inflammation, tissue edema, and inadequate drainage could potentially facilitate such extension. However, no direct anatomical connection was identified during surgery, and the microbiological findings did not provide evidence of a common infectious source: the perianal abscess culture showed no bacterial growth, whereas E. coli was isolated from the perineal abscess. Thus, the present findings do not establish direct transmission between the two abscesses. The two lesions may have developed independently, or they may have been influenced by local inflammatory processes that cannot be demonstrated retrospectively. Accordingly, local spread should be regarded as a possible mechanism rather than an established route of infection. Further cases with paired microbiological sampling and molecular or strain-level pathogen analysis may help clarify whether a microbiological relationship exists between these two conditions.

This case also highlights the importance of comprehensive anatomical assessment in patients with perineal infection. Bartholin gland and perianal abscesses can both present with pain, swelling, erythema, and tenderness, and severe cases may be accompanied by difficulty sitting or defecating and systemic inflammatory manifestations (3, 5, 7). Consequently, symptoms may initially be attributed to a single infectious focus, particularly when adjacent lesions are difficult to distinguish by physical examination. Differential diagnosis should include isolated vulvar soft-tissue abscess, Bartholin gland abscess, perianal abscess, and anal fistula (18). Persistent or progressive symptoms despite initial antimicrobial therapy should prompt reassessment of the extent and source of infection.

Imaging can complement physical examination by defining the location, depth, extent, and relationship of an abscess to surrounding structures. Ultrasonography is readily available and may be useful for evaluating superficial lesions (10, 19). For suspected deep infection or involvement of multiple adjacent anatomical compartments, pelvic MRI provides superior soft-tissue contrast and can better delineate the pelvic floor, perineal, and perianal structures, as well as possible residual abscesses and fistulous tracts (20). In the present case, CT was useful for identifying the initial extent of the inflammatory lesions and supporting the need for urgent drainage, whereas follow-up MRI provided additional anatomical information and subsequently demonstrated a cystic lesion in the left Bartholin gland region. This sequential use of imaging illustrates the value of reassessing the affected anatomical region when postoperative symptoms or persistent discharge cannot be fully explained by the initial diagnosis.

Once an abscess has formed, adequate drainage and source control remain the fundamental principles of treatment. For Bartholin gland abscesses, commonly used procedures include Word catheter placement, marsupialization, Jacobi ring fistulization, silver nitrate treatment, and gland excision (2, 3). Current evidence has not demonstrated consistent superiority of one gland-preserving procedure over another with respect to recurrence, although techniques that maintain a drainage tract may reduce the likelihood of premature closure of the abscess cavity (6, 9, 21, 22). The Jacobi ring technique used in the present case provided continuous drainage while preserving the surrounding vulvar tissue. The incision was made at the most dependent and fluctuant portion of the abscess at the skin–mucosal junction, facilitating drainage while limiting unnecessary tissue injury. The choice of procedure and incision site should nevertheless be individualized according to the size, location, and extent of the abscess, previous treatment history, and patient-specific factors (21).

Management of the perianal abscess requires a separate assessment of the anal gland and potential fistulous tract. In this patient, an infected anal gland was identified intraoperatively and excised, and bridge drainage was established between the 1- and 6-o'clock incisions. This approach was intended to provide adequate drainage of the extensive abscess cavity and reduce the risk of premature closure. Nevertheless, patients treated for perianal abscess remain at risk of recurrent abscess or subsequent anal fistula (7, 17). Follow-up should therefore include assessment for recurrent pain or swelling, persistent perianal discharge, or development of an external opening. In patients with recurrent or complex perianal abscesses, underlying inflammatory bowel disease should also be considered when clinically indicated.

This case has several strengths. First, it provides detailed clinical, radiological, and intraoperative information regarding the coexistence of Bartholin gland abscess and perianal abscess, a rarely reported clinical scenario. Second, the combination of imaging evaluation and direct surgical exploration allowed clarification of the anatomical relationship between the two abscesses and demonstrated the absence of a direct communication. Third, the involvement of both gynecological and anrectal surgical approaches enabled individualized management of the two separate infectious foci. These findings may provide practical insights into the diagnostic evaluation and surgical management of patients presenting with complex adjacent perineal infections.

The present case has several limitations. First, as a single case report, it cannot establish a causal relationship between Bartholin gland and perianal abscesses or determine whether the two infections arose independently or were biologically related. Second, although the perineal abscess yielded E. coli, the perianal specimen was culture-negative, and the available microbiological data were insufficient to determine whether the two lesions shared a common pathogen. Molecular pathogen detection or strain-level comparison was not performed. Third, the follow-up period was limited to 4 months, which is insufficient to fully assess long-term recurrence of Bartholin gland abscess, recurrent perianal abscess, or subsequent anal fistula formation.

In summary, this case demonstrates that Bartholin gland and perianal abscesses may coexist despite the absence of an identifiable communication between the two lesions. When adjacent perineal abscesses present with overlapping clinical manifestations, clinicians should avoid assuming a single infectious source and should systematically evaluate the vulvar, perineal, and perianal regions. Careful imaging assessment, intraoperative exploration, and timely collaboration between gynecology and anrectal surgery may facilitate accurate identification of multiple infectious foci and enable individualized surgical management. Further accumulation of similar cases is needed to clarify the potential anatomical and microbiological relationships between these uncommon concomitant infections.

## 4 Conclusion

The concomitant occurrence of a Bartholin gland abscess and a perianal abscess is uncommon and may pose a diagnostic challenge because of their overlapping clinical manifestations and close anatomical proximity. In patients with vulvar, perineal, or perianal abscesses, clinicians should consider the possibility of multiple infectious foci rather than assuming a single source, particularly when symptoms are extensive or fail to improve with initial treatment. Careful examination of adjacent anatomical regions, appropriate imaging, and intraoperative assessment can help define the extent and relationship of the lesions. When infections involve anatomically overlapping regions, timely multidisciplinary collaboration may facilitate accurate diagnosis and individualized surgical management. Long-term follow-up remains important because both Bartholin gland and perianal abscesses may recur or be complicated by fistula formation.

## 5 Patient perspective

Before the operation, the swelling and pain around my anus and perineum gradually worsened, while the diagnosis remained uncertain. After the doctor explained my condition and the proposed treatment plan to my mother and me, I decided to undergo surgery. During the first few days after the operation, there was still a considerable amount of discharge from the perineal area; however, after further examinations about one week later, my symptoms gradually improved. During follow-up, the wound around my anus had completely healed, although a small amount of discharge from the perineal area still occurred occasionally. Despite this, it did not interfere with my studies or daily life.

## Data Availability

The original contributions presented in the study are included in the article/Supplementary Material, further inquiries can be directed to the corresponding authors.
